# Antimetabolic Syndrome Effect of Phytosome Containing the Combined Extracts of Mulberry and Ginger in an Animal Model of Metabolic Syndrome

**DOI:** 10.1155/2019/5972575

**Published:** 2019-11-11

**Authors:** Nut Palachai, Jintanaporn Wattanathorn, Supaporn Muchimapura, Wipawee Thukham-mee

**Affiliations:** ^1^Department of Physiology and Graduate School (Neuroscience Program), Faculty of Medicine, Khon Kaen University, Khon Kaen 40002, Thailand; ^2^Integrative Complementary Alternative Medicine Research, Khon Kaen University, Khon Kaen 40002, Thailand; ^3^Research Institute for Human High Performance and Health Promotion, Khon Kaen University, Khon Kaen 40002, Thailand; ^4^Department of Physiology, Faculty of Medicine, Khon Kaen University, Khon Kaen 40002, Thailand

## Abstract

Due to the antimetabolic syndrome effect of mulberry and ginger together with the advantages of the synergistic effect and phytosome encapsulation technique, we hypothesized that phytosome containing the combined extracts of mulberry and ginger (PMG) should be able to manage MetS. PMG was developed and assessed the phenolic content and biological activities associated with the pathophysiology of MetS. The antimetabolic syndrome effect and the possible underlying mechanisms in the animal model of MetS were also assessed. Male Wistar rats induced MetS by subjecting to a 16-week high-carbohydrate high-fat diet. MetS rats were orally given PMG at doses of 50, 100, and 200 mg/kg for 21 days. They were determined metabolic parameter changes in serum, histomorphology changes of adipose tissue, the inflammatory cytokines such as IL-6 and TNF-*α*, oxidative stress status, PPAR-*γ*, and HDAC3 in adipose tissue. Our *in vitro* data showed that PMG increased phenolic contents and biological activities. PMG significantly improved MetS parameters including body weight gain, lipid profiles, plasma glucose, HOMA-IR, and ACE. In addition, the density and size of adipocyte, adiposity index, and weights of adipose tissues were also improved. Moreover, the decrease in TNF-*α* and IL-6, oxidative stress status, and HDAC3 expression together with the increase in PPAR-*γ* expression in adipose tissue was also observed. These data suggest that PMG exhibit antimetabolic syndrome and the possible underlying mechanism may be associated partly with the modulation effect on HDAC3, PPAR-*γ*, and adipose tissue. In addition, PMG also improves oxidative stress and inflammation in MetS. Therefore, PMG can be served as the potential supplement to manage MetS. However, a clinical trial study is essential to confirm this health benefit.

## 1. Introduction

Currently, the prevalence of metabolic syndrome (MetS), a complex disorder consisting of central obesity, hyperglycemia, hypertension, and hyperlipidemia [1], has been recognized as the global health problem [2]. MetS produces a great impact on the socioeconomic burden. In addition, it also produces numerous MetS-associated disorders including cardiovascular disorders and stroke. Despite its significance, the current therapy can successfully deal with some of the individual components such as hypertension, insulin resistance, and dyslipidemia. Unfortunately, such therapeutic success has not been shared by obesity, the other major component of the metabolic syndrome [3]. Therefore, the novel strategy that covers this component in MetS is still required.

Recent study has demonstrated that the alteration of adipose tissue plays a significant impact on whole-body metabolism and serves as a key driver for the development of these metabolic derangements [4]. The excess visceral tissue can induce numerous deleterious effects including insulin resistance, dyslipidemia, and inflammation [5]. Based on the key role of adipose tissue mentioned earlier, it has been considered as one of the treatment targets. Recently, it has been demonstrated that natural phytochemical substance possessing anti-inflammatory property such as capsaicin can improve MetS [6]. Other spice-derived components possessing an anti-inflammatory effect such as *Zingiber officinale* can also improve the aforementioned condition [7]. In addition to the inflammation, oxidative stress also plays an important role on the pathophysiology of MetS [8]. Substances possessing antioxidant activity such as anthocyanin-rich substances also exhibit an antimetabolic syndrome effect [8].

Most of the active ingredients of the medicinal plants, fruits, and vegetables are unstable and highly labile. Moreover, most of these phytochemical substances are poorly absorbed and instable during food processing, distribution, or storage in the gastrointestinal tract [9]. Therefore, a strategy to overcome all of these limitations is required. Interestingly, phytosome technology, a technology to conjugate phytochemicals to phospholipids in order to produce lipid compatible molecular complexes, is reported to improve the stability and bioavailability of the phytochemical substances [10–13]. It can improve stability by decreasing the decay induced by environment [14, 15]. Based on the advantages of a phytochemical substance in ginger and mulberry fruit together with the benefit of phytosome technology on stability and bioavailability mentioned earlier, we hypothesized that the phytosome containing the extract of mulberry and ginger could improve metabolic syndrome in metabolic syndrome rats. The changes of adipocyte, oxidative stress status, inflammation, PPAR-*γ*, and epigenetic mechanism were also explored to investigate the possible underlying mechanism.

## 2. Materials and Methods

### 2.1. PMG Preparation

Rhizomes of ginger (*Zingiber officinale* Roscoe) were collected from Khon Kaen province, Thailand, and authenticated by the expert in pharmacognosy of the National Museum of THAI Traditional Medicine, Thailand (voucher specimen No. 0002402 and deposited at the National Museum of THAI Traditional Medicine), and mulberry fruit (*Morus alba* Linn. var. Chiangmai) was identified and kindly provided by Mr. Sombat Kongpa, the chief of Queen Sirikit Department of Sericulture Center (Udon Thani Province), Ministry of Agriculture and Cooperatives, Thailand (voucher specimen 61001 and deposited at the Research Institute of Human High Performance and Health Promotion). The samples of both plants were cleaned and dried with the oven (Memmert GmbH, USA) at 60°C for 72 hours. Then, they were grounded to fine powder. Powder of ginger was prepared as 50% hydroalcoholic extract whereas mulberry powder was prepared as 95% hydroalcoholic extract by using maceration techniques. Then, the extracts were centrifuged at 3,000 rounds per minute (rpm) for 10 minutes and filtered with Whatman No. 1 filter paper. The filtrate was dried by using a rotator evaporator and freeze dryer.

According to the phytosome preparation, phosphatidylcholine was selected as encapsulation matrix. Mulberry extract and ginger extract were mixed at the ratio of 1 : 1 (*w*/*w*). The combined extract of mulberry and ginger was dissolved in 100 ml of 50% ethanol whereas phosphatidylcholine was dissolved in 50 ml of dichloromethane. Following this process, the solution was mixed together with magnetic stirring at 25°C for 8 hours. Then, ethanol and dichloromethane were removed by using a rotary evaporator at 45°C for 3 hours. The solution was frozen at -80°C overnight and dried by using lyophilization (Labconco freeze dryer, Labconco Corporation, Kansas City, MO, USA) for 48 hours (-86°C, 0.008 mbar). The dry sample was packed and stored in a desiccator containing silica gel at 4°C.

### 2.2. Determination of the Fingerprint Chromatogram

The fingerprint chromatogram of PMG was determined by using the high-performance liquid chromatography (HPLC) analysis. Chromatography was performed by using a Waters® system equipped with a Waters® 2998 photodiode array detector. The separation of chromatogram was performed using Purospher® STAR, C-18 encapped (5 *μ*m), LiChroCART® 250-4.6, and HPLC-Cartridge, Sorbet Lot No. HX255346 (Merck, Germany). According to this study, 100% methanol (solvent A) (Fisher Scientific, USA) and 2.5% acetic acid (solvent B) (Fisher Scientific, USA) in deionized (DI) water were used to produce the gradient of mobile phase. The gradient elution of mobile phase was carried out at a flow rate of 1.0 ml/min with the following gradient: 0-17 min, 70% A, 18-20 min, 100% A; 20.5-25 min, 10% A. The sample was filtered (0.45 *μ*m, Millipore) and an aliquot of sample at the volume of 20 *μ*l was directly administered. The chromatogram assessment was performed at 280 nm using a UV detector, and data analysis was performed using EmpowerTM3.

### 2.3. Measurement of Total Phenolic Compound Contents and Flavonoid Content

The amount of total phenolic compounds in a sample was determined by using the Folin-Ciocalteu colorimetric method in a microplate reader (iMark™ Microplate Absorbance Reader) [16].

The reagent consisting of 158 *μ*l of distilled water and 20 *μ*l of 50% *v*/*v* Folin-Ciocalteu reagent (Sigma-Aldrich, USA) was freshly prepared, mixed with 20 *μ*l of the extract, and incubated for 8 minutes. Following this process, 30 *μ*l of 20% Na_2_CO_3_ (Sigma-Aldrich, USA) was added and incubated at room temperature in a dark room for 2 hours. Then, an absorbance was measured at 765 nm. Result was expressed mg gallic acid equivalent (GAE)/mg extract. Various concentrations of gallic acid (Sigma-Aldrich, USA) were used as a standard calibration curve.

Flavonoid content was assessed by using the aluminum chloride method [17]. In brief, 100 *μ*l of PMG at various concentrations was mixed with 100 *μ*l of 2% methanolic aluminum chloride (Sigma-Aldrich, USA) and incubated at room temperature in a dark room for 30 minutes. At the end of incubation time, absorbance at 415 nm was measured against the suitable blank. Various concentrations of quercetin (Sigma-Aldrich, USA) were used for the standard calibration curve preparation. Results were expressed as *μ*g quercetin equivalent/mg extract.

### 2.4. Determination of Biological Activities

#### 2.4.1. Antioxidant Activity

Antioxidant activity of PMG was determined by using 1,1-diphenyl-2-picryl-hydrazyl (DPPH), ferric reducing antioxidant power (FRAP), and 2,2′-azinobis(3-ethylbenzothiazoline-6-sulphonic acid (ABTS) assays. According to DPPH assay, the stable DPPH assay was used for the determination of free radical-scavenging activity of PMG. In brief, 0.1 mM alcoholic solution of DPPH in methanol was prepared and 2 ml of this solution was mixed with 0.3 ml of PMG at various concentrations (1-100 mg/ml) and was allowed to react at 25°C. At the end of a 30-minute incubation time period, the absorbance value was measured at 517 nm against the blank via a microplate reader (iMark™ Microplate Absorbance Reader). L-Ascorbic acid was served as control. The radical-scavenging activity was expressed as percent inhibition of DPPH radical [18].

FRAP assay was carried out based on the ability of PMG to convert ferric tripyridyltriazine (Fe^3+^-TPTZ) to ferrous tripyridyltriazine (Fe^2+^-TPTZ). FRAP working solution was prepared by mixing 300 mM acetate buffer (Sigma-Aldrich, USA), 10 mM TPTZ (Sigma-Aldrich, USA), and 20 mM ferric chloride (FeCl_3_) (Sigma-Aldrich, USA) solutions at a ratio of 10 : 1 : 1, respectively. In brief, 190 *μ*l of FRAP reagent was mixed with 10 *μ*l of PMG and incubated at 37°C for 10 minutes. After the incubation time period, an absorbance at 593 nm was measured against blank [19]. Vitamin C was used as a positive control, and results were as expressed as EC_50_ value.

ABTS assay was also used to determine the free radical-scavenging activity of the PMG [20]. ABTS^+^ solution was prepared by mixing the 7 mM ABTS (Sigma-Aldrich, USA) and 2.45 mM potassium persulfate (K_2_S_2_O_8_) (Sigma-Aldrich, USA) at a ratio of 2 : 3. Then, 3 ml of the solution was reacted with 120 *μ*l of distilled water, 30 *μ*l of ethanol, and 30 *μ*l of various concentrations of PMG. The absorbance was measured at 734 nm with a spectrophotometer (Pharmacia LKB-Biochrom 4060). Trolox was used as a positive control. Results were also expressed as EC_50_ value.

#### 2.4.2. Assessment of Pancreatic Lipase

The working solution of lipase at a concentration of 10 ng/ml was prepared by dissolving lipase from porcine pancreas type II (Sigma, USA) in deionized (DI) water and centrifuged to a 16,000 rpm centrifugation for 5 minutes. Then, the supernatant was harvested for further used. In brief, 100 mM Tris buffer pH 8.2 and p-nitrophenyl laurate (pNP laurate) were used as the substrate. The pNP laurate was dissolved in 5 mM sodium acetate (pH 5.0) containing 1% Triton X-100 to produce 0.08% *w*/*v* substrate solution and served as stock solution. This solution was heated in boiling water for 1 min to aid dissolution, mixed well, and then cooled to 25°C. The reaction mixture containing 70 *μ*l of assay buffer, 90 *μ*l of substrate solution, 30 *μ*l of lipase, and 10 *μ*l of PMG was mixed and incubated at 37°C for 2 hours. At the end of the incubation time period, the solution was centrifuged at 16,000 rpm for 1 minute and measured absorbance with a microplate reader (iMark™ Microplate Absorbance Reader) at 400 nm. [21]. The orlistat was used as a positive control.

#### 2.4.3. Assessment of Cyclooxygenase-2 (COX-2)

COX-2 inhibition was measured by using a colorimetric COX-2 inhibitor screening assay kit (COX Activity Assay Kit, item No.760151) (Cayman Chemical, USA). The effect of PMG on COX-2 inhibition activity was performed according to the manufacturer's protocol. Cox-2 working solution was prepared by dissolving COX-2 agent in 100 mM Tris-HCl buffer, pH 8.0 at a ratio of 1 : 100. In brief, the reaction mixture containing 150 *μ*l of assay buffer, 10 *μ*l of PMG, 10 *μ*l of heme (Cayman Chemical, USA), 10 *μ*l of COX-II working solution, 20 *μ*l of 10 *μ*M TMPD (N,N,N′,N′-tetramethyl-p-phenylenediamine dihydrochloride) (Sigma, USA), and 20 *μ*l of 100 *μ*M arachidonic acid (Cayman Chemical, USA) was added to a 96-well microplates and incubated at 25°C for 30 minutes. At the end of the incubation time period, an absorbance at 590 nm was recorded and results were expressed as EC_50_ [22]. Indomethacin was used as a positive control.

### 2.5. Experimental Protocol

Male Wistar rats (weighting 180-220 g, 8 weeks old) were obtained from the National Laboratory Animal Center, Salaya, Nakhon Pathom, Thailand. The rats were kept under standard laboratory conditions with food and water ad libitum and housed in standard metal cages (6 per cage). Temperature was controlled at 23 ± 2°C on a 12 : 12 hour light-dark cycle. All procedures and experimental protocols were approved by the Institutional Animal Ethics Committee of Khon Kaen University (record no. IACUC-KKU 95/60). After 1 week of acclimatization, rats were divided into 7 groups (*n* = 6) as follows:

1. Group I (ND+vehicle): all rats in this group were administered normal diet and treated with vehicle
2. Group II (HCHF+vehicle): all rats in this group received high-carbohydrate high-fat (HCHF) diet and treated with vehicle
3. Group III (HCHF diet+vitamin C): all rats in this group received HCHF diet and treated with vitamin C at a dose of 250 mg/kg BW
4. Group IV (HCHF diet+simvastatin): animals in this group received HCHF diet and treated with simvastatin at a dose of 1.3 mg/kg BW
5. Group V-VII (HCHF diet+PMG) (PMG50, PMG100, and PMG 200): all rats in these groups received HCHF diet and treated with PMG at various doses ranging from 50 and 100 to 200 mg/kg BW

**Figure 1:**
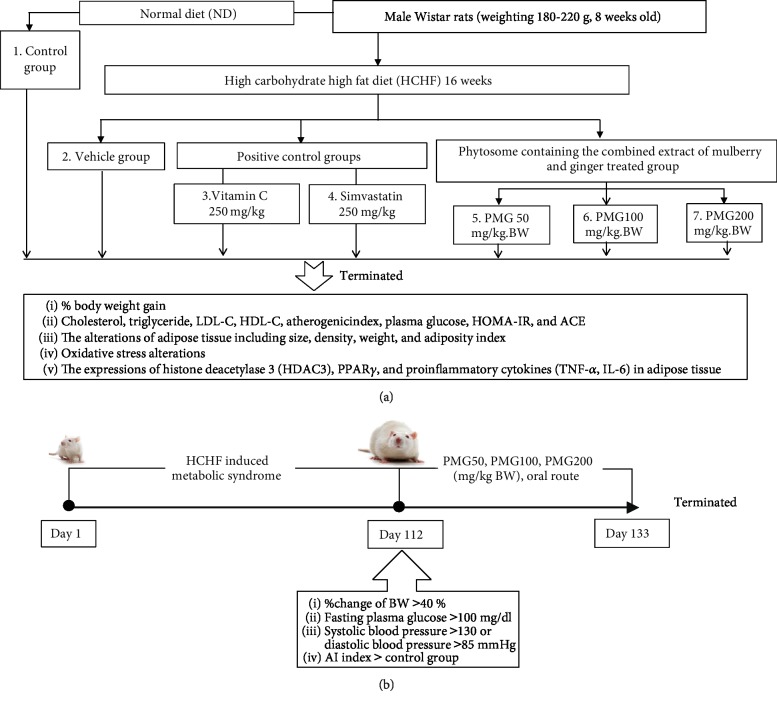
Schematic diagram showing all experimental procedures. (a) Experimental protocol of PMG treatment and the determination of various parameters. (b) Metabolic syndrome induction and schedule for PMG treatment. LDL-C: low-density lipoprotein cholesterol; HDL-C: high-density lipoprotein cholesterol; HOMA-IR: homeostasis model assessment of insulin resistance; ACE: angiotensin-converting enzyme; HDAC3: histone deacetylase 3; PPAR-*γ*: peroxisome proliferator-activated receptor gamma; IL-6: interleukin-6; TNF-*α*: tumor necrosis factor-*α*; PMG50, PMG100, and PMG200: the phytosome containing the combined extracts of mulberry and ginger at a dose of 50, 100, and 200 mg·kg^−1^ BW, respectively.

Rats in group I were fed with normal diet (4.5% fat, 42% carbohydrate, and 24% protein) whereas rats in group II-VII were fed with HCHF diet (HCHF; 35% fat, 45% carbohydrate, and 20% protein) in order to induce metabolic syndrome. After 16 weeks of the feeding period, rats which showed the percentage change of body weight more than 40 percent, fasting plasma glucose more than 100 mg/dl, systolic blood pressure more than 130 or diastolic blood pressure more than 85 mmHg, and the atherosclerosis index (total serum cholesterol/total serum HDL-C) higher than the control group were selected for further study. Then, the animals were orally given the assigned substances once daily for 21 days. Food and water consumption together with body weight was assessed every day throughout the study period. At the end of the study period, %body weight gain, cholesterol, triglyceride, LDL-C, HDL-C, atherogenic index, plasma glucose, HOMA-IR, and ACE were determined. In addition, the alterations of adipose tissue including size, density, weight, and adiposity index together with the oxidative stress status, the expressions of histone deacetylase 3 (HDAC3), PPAR-*γ*, and proinflammatory cytokines (TNF-*α*, IL-6) in adipose tissue were also determined. The schematic diagram showing the experimental protocol is shown in Figure 1.

### 2.6. Biochemical Assay

#### 2.6.1. Plasma Lipid Profiles

*(1) Determination of Total Plasma Cholesterol*. The total plasma cholesterol was determined by “CHOD-PAP”: enzymatic photometric test [23]. 10 *μ*l of plasma or calibrator was mixed with 1,000 *μ*l of cholesterol FS Reagent (DiaSys Diagnostic Systems GmbH, Germany) and incubated at 25°C in a dark room for 20 minutes. Then, an absorbance at 500 nm was measured within 60 minutes by using a UV-spectrophotometer (Pharmacia LKB-Biochrom 4060). Total cholesterol was expressed as mg/dl and calculated as follows:

(1) $$\begin{array}{c} \text{Cholesterol }\left(\text{mg}/\text{dl}\right)=\left(\frac{A\text{ sample}}{A\text{ calibrator}}\right)\times \text{Conc}.\text{Cal}.\left(\text{mg}/\text{dl}\right). \end{array}$$

*(2) Determination of Plasma Triglycerides*. The enzymatic colorimetric test was performed by using glycerol-3-phosphate-oxidase (GPO) reagent (DiaSys Diagnostic Systems GmbH, Germany). 10 *μ*l of plasma or calibrator was mixed with 1,000 *μ*l of the prepared reagent and incubated at 25°C in a dark room for 10 minutes. The absorbance was measured at 500 nm within 60 minutes by using a UV-spectrophotometer (Pharmacia LKB-Biochrom 4060). Triglycerides was expressed as mg/dl and calculated as follows:

(2) $$\begin{array}{c} \text{Triglycerides }\left(\text{mg}/\text{dl}\right)=\left(\frac{A\text{ sample}}{A\text{ calibrator}}\right)\times \text{Conc}.\text{Cal}.\left(\text{mg}/\text{dl}\right). \end{array}$$

*(3) Determination of Plasma Low-Density Lipoprotein Cholesterol (LDL-C)*. Plasma LDL-C was assessed based on the Friedewald equation [24] by using LDL-C select FS Reagent 1 and 2 (DiaSys Diagnostic Systems GmbH, Germany). In brief, 5 *μ*l of plasma or Trulab L calibrator and 280 *μ*l of reagent 1 were mixed and incubated at 37°C for 5 minutes. After the incubation time period, an absorbance at 595 nm (A1) was measured by using a microplate reader (iMark™ Microplate Absorbance Reader). Then, 70 *μ*l of reagent 2 was added to the mixture and incubated at 37°C for 5 minutes. After that, the absorbance at 595 nm (A2) was measured. LDL-C was expressed as mg/dl and calculated as follows:

(3) $$\begin{array}{c} \text{LDL}‐\text{C }\left(\text{mg}/\text{dl}\right)=\left(\frac{A\text{ sample}}{A\text{ calibrator}}\right)\times \text{Conc}.\text{Cal}.\left(\text{mg}/\text{dl}\right),\\ \text{A}=\left[\left(\text{A}2-\text{A}1\right)\text{ sample or calibrator}\right]-\left[\left(\text{A}2-\text{A}1\right)\text{ blank}\right] \end{array}$$

*(4) Determination of Plasma High-Density Lipoprotein Cholesterol (HDL-C)*. Plasma HDL was determined by using HDL-C select FS Reagent 1 and 2 (DiaSys Diagnostic Systems GmbH, Germany) [25]. In brief, 5 *μ*l of plasma or Trulab L calibrator and 240 *μ*l of reagent 1 were mixed and incubated at 37°C for 5 minutes. After the incubation time period, an absorbance at 595 nm (A1) was measured by using a microplate reader (iMark™ Microplate Absorbance Reader). Then, 60 *μ*l of reagent 2 was added to the mixture and incubated at 37°C for 5 minutes. After the incubation, the absorbance at 595 nm (A2) was measured. HDL-C was expressed as mg/dl and calculated as follows:

(4) $$\begin{array}{c} \text{HDL}‐\text{C }\left(\text{mg}/\text{dl}\right)=\left(\frac{A\text{ sample}}{A\text{ calibrator}}\right)\times \text{Conc}.\text{Cal}.\left(\text{mg}/\text{dl}\right),\\ \text{A}=\left(\text{A}2-\text{A}1\right)\text{ sample or calibrator} \end{array}$$

#### 2.6.2. Estimation of Atherogenic Index (AI Index)

The atherogenic index (AI index), the most reliable indicator for the prediction of cardiovascular disease risk, was determined and calculated according to the following equation [26]:

(5) $$\begin{array}{c} \text{Atherogenic index }\left(\text{AI index}\right)=\frac{\text{total cholesterol }\left(\text{TC}\right)}{\text{high}‐\text{density lipoprotein cholesterol }\left(\text{HDL}‐\text{C}\right)\text{ ratio}}. \end{array}$$

#### 2.6.3. Estimation of Plasma Glucose

All animals were fasted for 12 hours. After the food deprivation period, the basal blood glucose concentrations were measured. In brief, the blood sample was collected from tail vein and determined the blood glucose level by using ACCU-CHEK® Performa Blood Glucose Meter.

#### 2.6.4. Estimation of HOMA-IR

At the end of a 12-hour fasting period, serum insulin and plasma glucose determinations were performed in order to calculate the homeostasis model assessment of insulin resistance (HOMA-IR) [27]. Blood samples were collected and immediately kept cool in ice bath. Then, they were centrifuged at 1,000 x g for 10 minutes at room temperature, according to the manufacturer's instructions. Serum was stored at -80°C until the assay. Insulin assay was performed using the Luminex™ kit (Millipore™, Billerica, MA) following the provided luminescence method. HOMA-IR was calculated according to the following equation:

(6) $$\begin{array}{c} \text{HOMA IR}=\frac{\text{serum insulin }\left(\text{mmol}/\text{l}\right)\times \text{blood glucose }\left(\text{mmol}/\text{l}\right)}{22.5\text{ insulin assay}}. \end{array}$$

#### 2.6.5. Determination of Plasma Angiotensin-Converting Enzyme

Plasma angiotensin-converting enzyme was performed base on Serra et al.'s concept [28]. The enzymatic reaction was started by adding the 20 *μ*l of plasma into the 50 *μ*l of substrate solution Hip–Gly–Gly (100 mmol/l) (Sigma, USA) and incubated at 37°C for 35 minutes. The reaction was stopped by adding 120 *μ*l of 3 M sodium tungstate (Sigma, USA) and 0.5 M sulfuric acid (Sigma, USA) and centrifuged at 2500 rpm for 10 minutes. After the centrifugation, the supernatant was placed into a 96-well microplate and mixed with 20 *μ*l of 60 mM TNBS (Sigma, USA) and incubated at dark condition for 20 minutes. At the end of the incubation time period, an absorbance at 415 nm was recorded with a microplate reader (iMark™ Microplate Absorbance Reader). The standard calibration curve was prepared by using ACE (Sigma-Aldrich, USA) at the concentration range of 0.001-1 units/ml. Results were expressed as units/mg protein.

### 2.7. Histological Procedure and Adiposity Assessment

After the scarification, fat pads from visceral and subcutaneous areas were removed and immersed into fixative solution containing 10% formalin (Sigma-Aldrich, USA) for 72 hours. Serial sections of tissues were cut frozen on cryostat (Thermo Scientific™ HM 525 Cryostat) at 10 *μ*m thick. All sections were picked up on slides coated with 0.3% aqueous solution of gelatin containing 0.05% aluminum potassium sulfate (Sigma-Aldrich, USA). The observation was carried out after the hematoxylin-eosin (H&E) (Sigma-Aldrich, USA) staining process [29]. The determination of adipocyte cell diameter and density were performed from 3 randomly selected different fields of each area by using Olympus light microscope model BH-2 (Japan) under 40x magnification with PixeLINK PL-A6xx Capture and IT tool program. In addition, the adiposity index was calculated by the sum of intra‐abdominal fat weights/body weight ratio × 100 and expressed as percentage of adiposity.

### 2.8. Assessment of Oxidative Stress Status in Adipose Tissues and Plasma

Adipose tissues were isolated and homogenized with 0.1 M potassium phosphate buffer solution, pH 7.4 (sample dilution 10 mg: PBS 50 *μ*l). The derived homogenate was used for the determination of oxidative status, including the activities of superoxide dismutase (SOD), catalase (CAT), glutathione peroxidase (GSH-Px), and malondialdehyde (MDA) level. The protein concentrations in adipose tissue homogenate were assessed by using a Thermo Scientific NanoDrop 2000c spectrophotometer (Thermo Fisher Scientific, Wilmington, Delaware, USA) and measured the optical density at the wavelength of 280 nm.

According to assess SOD activity, the reaction solution was prepared by mixing the 0.2 M phosphate buffer solution (KH_2_PO_4_), pH 7.8 (Sigma-Aldrich, USA), 0.01 M EDTA (Sigma-Aldrich, USA), 15 M cytochrome C (Sigma-Aldrich, USA), and 0.5 mM of xanthine, pH 7.4 (Sigma-Aldrich, USA) together at the ratio of 25 : 1 : 1 : 50 (*v*/*v*). 20 *μ*l of tissue homogenate was mixed with 200 *μ*l of the reaction mixture and 20 *μ*l of xanthine oxidase (0.90 mU/ml) (Sigma-Aldrich, USA). The optical density at 415 nm was measured. SOD enzyme (Sigma-Aldrich, USA) activities at the concentrations of 0-25 units/ml were used as standard, and the results were expressed as units/mg protein [30].

Base on the capability of the enzyme to break down, H_2_O_2_ was used for determining catalase activity. In brief, the reaction mixture containing 50 *μ*l of 30 mM hydrogen peroxide (in 50 mM phosphate buffer, pH 7.0) (BDH Chemicals Ltd., UK), 25 *μ*l of 5 M H_2_SO_4_ (Sigma-Aldrich, USA), and 150 *μ*l of 5 mM KMnO_4_ (Sigma-Aldrich, USA) was mixed with 10 *μ*l of sample. After mixing, an absorbance at 490 nm was recorded [31]. CAT enzyme (Sigma-Aldrich, USA) at the concentration range between 10 and 100 units/ml was used as standard, and the result was expressed as units/mg protein.

Glutathione peroxidase activity was assessed by mixing 20 *μ*l of sample solution with the reaction mixture consisting of 10 *μ*l of 1 mM dithiothreitol (DTT) (Sigma-Aldrich, USA) and 10 mM monosodium phosphate (NaH_2_PO_4_) in DW, 100 *μ*l of 1 mM sodium azide (Sigma-Aldrich, USA) in 40 mM potassium phosphate buffer (pH 7.0), 10 *μ*l of 50 mM glutathione (Sigma-Aldrich, USA) solution, and 100 *μ*l of 30% hydrogen peroxide (BDH Chemicals Ltd., UK) and incubated at 25°C for 10 minutes. At the end of the incubation time period, 10 *μ*l of 10 mM DTNB (5,5-dithiobis-2-nitrobenzoic acid) (Sigma-Aldrich, USA) was added and an absorbance at 412 nm was recorded [32]. GSH-Px enzyme (Sigma-Aldrich, USA) at the concentration range between 1 and 5 units/ml was used as standard. GSH-Px activity was expressed as units/mg protein.

The MDA level was also assessed according to the thiobarbituric acid reaction method [33]. The reaction mixture containing 50 *μ*l of sample solution, 50 *μ*l of 8.1% sodium dodecyl sulfate (SDS) (Sigma-Aldrich, USA), 375 *μ*l of 0.8% of thiobarbituric acid (TBA) (Sigma-Aldrich, USA), 375 *μ*l of 20% acetic acid (Sigma-Aldrich, USA), and 150 *μ*l of distilled water (DW) was boiled at 95°C in the water bath for 60 minutes. After boiling, it was cooled with tap water. After that, 250 *μ*l of DW and 1,250 *μ*l of the solution containing n-butanol and pyridine (Merck, Germany) at the ratio of 15 : 1 were added, mixed together, and centrifuged at 4,000 rpm for 10 minutes. The upper layer was separated and measured the absorbance at 532 nm. TMP (1,1,3,3-tetramethoxypropane) (0-15 *μ*M) (Sigma-Aldrich, USA) was served as standard, and the level of MDA was expressed as ng/mg protein.

### 2.9. Western Blotting Analysis

Adipose tissue was homogenized and lysed in 1/5 (*w*/*v*) RIPA (radioimmunoprecipitation assay) buffer (Cell Signaling Technology, USA) containing 20 mM Tris-HCl (pH 7.5), 150 mM NaCl, 1 mM Na2EDTA, 1 mM EGTA, 1% NP-40, 1% sodium deoxycholate, 2.5 mM sodium pyrophosphate, 1 mM beta-glycerophosphate, 1 mM Na3VO4, 1 *μ*g/ml leupeptin, and 1 mM phenylmethanesulfonyl fluoride (PMSF) (Cell Signaling Technology, USA). The tissue homogenate supernatant of the middle layer of adipose tissue samples was isolated after the 12,000 g centrifugation at 4°C for 10 minutes. Protein concentration was determined by using a Thermo Scientific NanoDrop 2000c spectrophotometer (Thermo Fisher Scientific, Wilmington, Delaware, USA). In brief, 80 micrograms of tissue lysates were adjusted to appropriate concentration by using Tris-Glycine SDS-PAGE loading buffer and heated at 95°C for 10 minutes. Protein in tissue sample was isolated via sodium dodecyl sulfate-polyacrylamide gel electrophoresis (SDS-PAGE) by loading 20 *μ*l of sample on SDS-polyacrylamide gel. Then, the separated bands were transferred to nitrocellulose membrane, washed with 0.05% TBS-T, and incubated in blocking buffer (5% skim milk in 0.1% TBS-T) at 25°C for 1 hour. After the blocking process, the nitrocellulose membrane was incubated with anti-HDAC3 (Cell Signaling Technology, USA; dilution 1 : 500), anti-PPAR gamma (Abcam, UK; dilution 1 : 1000), anti-IL-6 (Cell Signaling Technology, USA; dilution 1 : 500), anti-TNF-*α* (Cell Signaling Technology, USA; dilution 1 : 500), and anti-*β*-actin (Cell Signaling Technology, USA; dilution 1 : 1000) antibodies at 4°C overnight. After an incubation time period, the nitrocellulose membrane was rinsed with TBS-T (0.05%) again and incubated with anti-rabbit IgG, HRP-linked antibody (Cell Signaling Technology, USA; dilution 1 : 2000) at 25°C for 1 hour. The bands were visualized and quantitated by using the ECL detection systems (GE Healthcare) and LAS-4000 luminescent image analyzer (GE Healthcare). Band intensities were measured for statistical analysis using ImageQuant TL v.7.0 image analysis software (GE Healthcare). The expression was normalized using anti-*β*-actin. Data were presented as a relative density to the naïve control group [34].

### 2.10. Statistical Analysis

All data are expressed as mean ± standard error of mean (SEM). Statistical significance was evaluated by using one-way analysis of variance (ANOVA), followed by the post hoc (Tukey) test. Student's *t* test was used for comparison of the means for two groups. Statistical significance was regarded at *p* values < 0.05. All statistical data analyses were performed using SPSS version 21.0 (IBM Corp. Released 2012. IBM SPSS Statistics for Windows).

## 3. Results

### 3.1. Fingerprint Chromatogram and the Determination of Phenolic Compounds, Flavonoids, and Biological Activities

**Figure 2:**
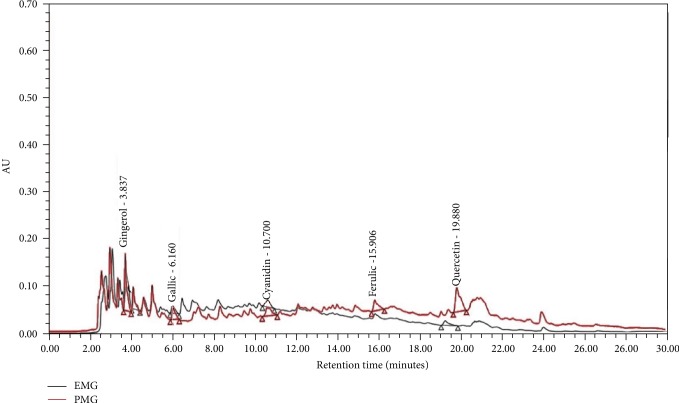
The HPLC profiles of gingerol, gallic acid, cyanidin-3-glucoside, ferulic acid, and quercetin-3-O-rutinoside of EMG and PMG. EMG: the combined extracts of mulberry and ginger; PMG: the phytosome containing the combined extracts of mulberry and ginger.

Figure 2 shows the fingerprint chromatogram of EMG and PMG whereas Table 1 shows the contents of phenolic compounds, flavonoids, and the important active ingredients in the combined extracts of mulberry and ginger (EMG) and phytosome containing the combined extracts of mulberry and ginger (PMG). Both EMG and PMG showed the similar chromatograms but differed in the concentration of the ingredients. It was found that EMG contained total phenolic and flavonoid contents at the concentrations of 243.00 ± 30.55 mg GAE/mg extract and 90.33 ± 15.40 *μ*g quercetin/mg extract, respectively. It was found that each 50 mg of EMG contained gingerol, cyanidin-3-O-glucoside, quercetin-3-rutinoside, ferulic acid, and gallic acid at the concentrations of 9.45 ± 0.03 *μ*g gingerol, 11.63 ± 0.06 *μ*g Cyn-3-glu, 25.93 ± 0.17 *μ*g Rutin, 12.89 ± 0.42 *μ*g ferulic acid, and 11.26 ± 0.01 *μ*g GAE. However, the contents of total phenolic, flavonoids, gingerol, cyanidin-3-O-glucoside, quercetin-3-rutinoside, ferulic acid, and gallic acid in PMG were 273.00 ± 5.77 mg GAE/mg extract, 137.00 ± 3.85 *μ*g quercetin/mg extract, 19.23 ± 0.03 *μ*g gingerol/50 mg extract, 16.01 ± 0.06 *μ*g Cyn-3-glu/50 mg extract, 39.82 ± 0.40 *μ*g Rutin/50 mg extract, 20.00 ± 0.18 *μ*g ferulic acid/50 mg extract, and 47.89 ± 0.16 *μ*g GAE/50 mg extract, respectively. These data clearly revealed that PMG contained all substance mentioned earlier higher than EMG (*p* value < 0.05, 0.001, 0.001, 0.01, 0.05, and 0.001) except total polyphenolic compound.

The biological activities associated with the pathophysiology of MetS such as antioxidant, antiobesity, and anti-inflammation were also assessed. The EC_50_ of an antioxidant effect determined by DPPH assay of EMG and PMG were 39.97 ± 1.68 and 39.67 ± 0.93 mg/ml whereas EC_50_ which derived from FRAP were 97.15 ± 3.17 and 41.28 ± 0.37 and EC_50_ of both substances from ABTS assay were 58.46 ± 0.66 and 47.28 ± 1.89 mg/ml. The suppression activities of pancreatic lipase and COX-II were also determined. EC_50_ values of the suppression activity of pancreatic lipase of EMG and PMG were 181.56 ± 17.87 and 138.15 ± 0.92 mg/ml. It was found that the EC_50_ value of COX-II suppression effect of EMG and PMG was 81.25 ± 2.97 and 80.42 ± 1.85 mg/ml. The current data showed that the PMG exhibited more potent antioxidant activity via FRAP and ABTS and more potent activity of pancreatic lipase suppression activity than EMG (*p* value < 0.001, 0.01, and 0.01, respectively).

### 3.2. Antimetabolic Syndrome Effect Assessment

Table 2 showed the effect of PMG at various doses on metabolic parameters. HCHF diet significantly increased the percent of body weight gain, triglyceride, cholesterol, LDL-C, blood glucose, HOMA-IR, AI-index, and ACE activity but decreased HDL-C (*p* value < 0.05, 0.01, 0.001, 0.001, 0.01, 0.001, 0.01, 0.001, and 0.001, respectively; compared to naïve control). Vitamin C failed to show the positive modulation effects on the aforementioned parameters. Simvastatin and PMG at doses of 50 and 100 mg/kg BW significantly decreased the percent of body weight gain, triglyceride, cholesterol, LDL-C, AI-index, and ACE but increased HDL-C (*p* value < 0.01, 0.001, and 0.001; *p* value < 0.001 all; *p* value < 0.05, 0.01, and 0.01; *p* value < 0.001 all; *p* value < 0.01, 0.001, and 0.001; *p* value < 0.01, 0.001, and 0.001; *p* value < 0.05 all; compared to the HCHF diet-treated group). All doses of PMG could significantly improve HOMA-IR (*p* value < 0.05, 0.01, and 0.05, respectively; compared to HCHF diet-treated group). Interestingly, PMG at a dose of 200 mg/kg BW significantly decreased body weight gain, cholesterol, triglyceride, LDL-C, AI-index, plasma glucose, HOMA-IR, and ACE but increased HDL-C (*p* value < 0.001, 0.01, 0.001, 0.001, 0.001, 0.05, 0.05, 0.001, and 0.01, respectively; compared to HCHF diet-treated group).

### 3.3. Changes of Adipose Tissue

**Figure 3:**
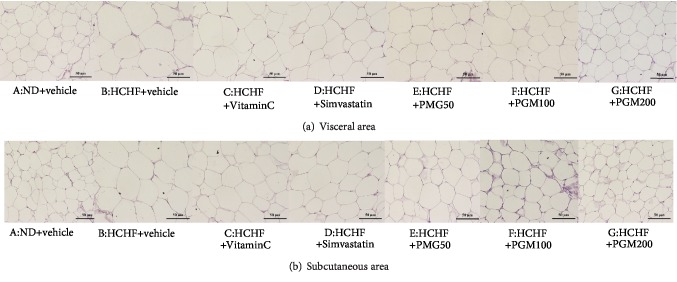
Light microscope of white adipose tissue was stained with hematoxylin and eosin (H&E) at 40x magnification. ND: normal diet; HCHF: high-carbohydrate high-fat diet; VitaminC: vitamin C at a dose of 250 mg·kg^−1^ BW; Simvastatin: simvastatin at a dose of 1.3 mg·kg^−1^ BW; PMG50, PMG100, and PMG200: the phytosome containing the combined extracts of mulberry and ginger at a dose of 50, 100, and 200 mg·kg^−1^ BW, respectively.

The effect of PMG on the weight of adipose tissue, size, and density of adipocyte together with adiposity index in both visceral and subcutaneous areas was determined, and data are shown in Figure 3 and Table 3. MetS rats showed the increase in weight of adipose tissue and size of adipocyte in both areas mentioned earlier (*p* value < 0.001 and 0.01; *p* value < 0.001 all; compared to the naïve control group). An adiposity index in the visceral area also increased (*p* value < 0.001; compared to the naïve control group). In addition, the density of adipocyte of MetS rats also decreased (*p* value < 0.001 all; compared to the naïve control group). Vitamin C failed to produce the significant changes of all parameters just mentioned in MetS rats. Simvastatin decreased the size but increased the density of adipocyte in the visceral area (*p* value < 0.05 and 0.01, respectively; compared to the HCHF-treated group). All doses of PMG significantly decreased the size of adipocyte in visceral and subcutaneous areas (*p* value 0 < 0.001 all; *p* value 0 < 0.01, 0.01, and 0.001, respectively; compared to the HCHF-treated group) but increased the density of adipocyte in the areas just mentioned (*p* value < 0.001 all; *p* value < 0.05, 0.001, and 0.001, respectively; compared to the HCHF-treated group). In addition, the decrease in weight of adipose tissue and adiposity index in the visceral area was also observed in MetS rats which received PMG at doses of 100 and 200 mg/kg BW (*p* value < 0.001 all; compared to the HCHF-treated group).

### 3.4. Oxidative Stress Changes

The effect of PMG on oxidative stress markers in both visceral and subcutaneous areas together with oxidative stress markers in plasma is shown in Tables 4–6. HCHF diet significantly increased the MDA level in both visceral and subcutaneous areas of adipose tissue, and plasma of normal rats (*p* value < 0.001 all, compared to normal rats, which received normal diet and vehicle). Vitamin C and all doses of PMG significantly mitigated the elevation of the MDA level in both visceral and subcutaneous areas of adipose tissue, and plasma of metabolic syndrome rats, which fed with HCHF diet (*p* value < 0.001 all, compared to the HCHF-treated group). MetS rats induced by HCHF diet significantly decreased SOD activities in adipose tissue of both visceral and subcutaneous areas and in plasma (*p* value < 0.001 all; compared to naïve control). These changes were mitigated by vitamin C and PMG at doses of 50, 100, and 200 mg/kg in the visceral area (*p* value < 0.001, 0.001, 0.001, and 0.01, respectively, compared to the HCHF diet-treated group), subcutaneous area (*p* value < 0.05, 0.001, 0.001, and 0.001, respectively, compared to the HCHF diet-treated group), and plasma (*p* value < 0.001 all, compared to the HCHF diet-treated group).

In addition, metabolic syndrome rats induced by HCHF diet showed the significant decrease in CAT activity in adipose tissue of both visceral and subcutaneous areas and in plasma (*p* value < 0.001 all, compared to naïve control). However, vitamin C and PMG at doses of 100 and 200 mg/kg significantly increased CAT activity in the visceral area (*p* value < 0.01, 0.05, and 0.05, respectively, compared to HCHF diet-treated group). Moreover, vitamin C and PMG at all doses significantly increased CAT activity in the subcutaneous area (*p* value < 0.01 all, compared to the HCHF diet-treated group). Only PMG treatment can produce the significant increased CAT activity in serum (*p* value < 0.001 all, compared to the HCHF diet-treated group).

It was also found that HCHF diet produced the significant decrease in GSH-Px activity in adipose tissue of both visceral and subcutaneous areas and in plasma of normal rats (*p* value < 0.001 all, compared to naïve control). However, vitamin C and all doses of PMG could attenuate the change of this parameter in both visceral and subcutaneous areas (*p* value < 0.001 all, compared to the HCHF diet-treated group) and in plasma (*p* value < 0.05, 0.001, 0.001, and 0.001, respectively, compared to the HCHF diet-treated group).

### 3.5. Histone Deacetylase 3 (HDAC3) Expression Change

**Figure 4:**
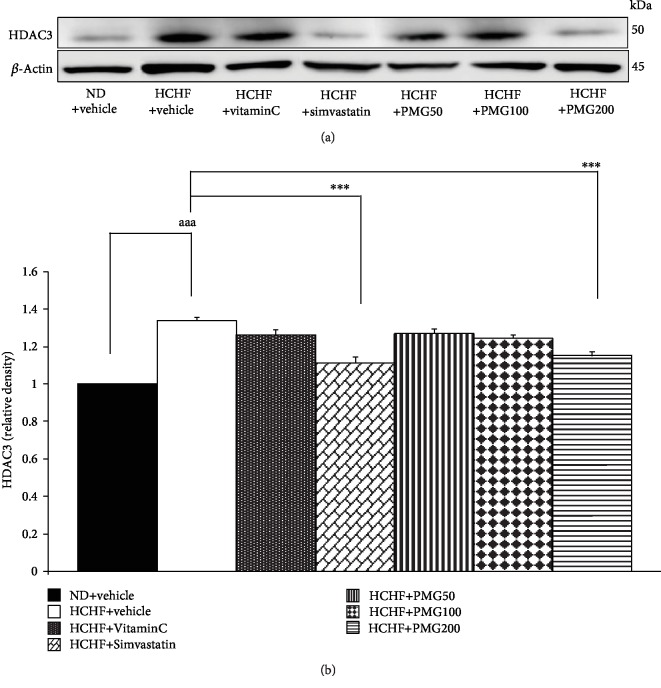
Effect of various doses of PMG on the expression of HDAC3 in adipose tissue. (a) Representative western blot showing the levels of HDAC3. (b) Relative density of HDAC3. Data are presented as mean ± SEM (*n* = 6/group). ^aaa^*p* value < 0.001, compared to naïve control which received ND and vehicle and ^∗∗∗^*p* value < 0.001, compared to metabolic syndrome rats which received HCHF and vehicle. ND: normal diet; HCHF: high-carbohydrate high-fat diet; VitaminC: vitamin C at a dose of 250 mg·kg^−1^ BW; Simvastatin: simvastatin at a dose of 1.3 mg·kg^−1^ BW; PMG50, PMG100, and PMG200: the phytosome containing the combined extracts of mulberry and ginger at a dose of 50, 100, and 200 mg·kg^−1^ BW, respectively.

Effect of PMG on the expression of HDAC3 in adipose tissue was also determined, and results are shown in Figure 4. MetS rats which received HCHF significantly increased HDAC3 expression in adipose tissue (*p* value < 0.001, compared to naïve control). Simvastatin and PMG at a high dose significantly decreased HDAC3 expression in adipose tissue (*p* value < 0.001 all, compared to the HCHF diet-treated group).

### 3.6. Effect of PMG on PPAR-*γ* Expression

**Figure 5:**
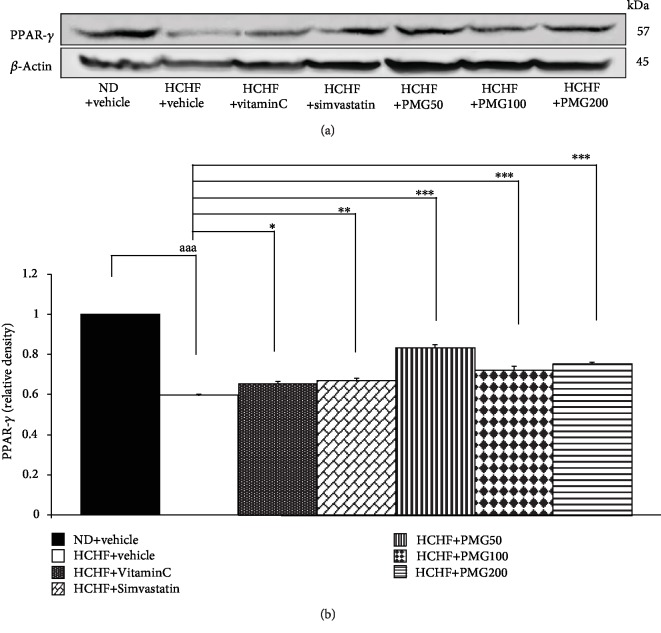
Effect of various doses of PMG on the expression of PPAR-*γ* in adipose tissue. (a) Representative western blot showing the levels of PPAR-*γ*. (b) Relative density of PPAR-*γ*. Data are presented as mean ± SEM (*n* = 6/group). ^aaa^*p* value < 0.001, compared to naïve control which received ND and vehicle and ^∗,∗∗,∗∗∗^*p* value < 0.05, 0.01, and 0.001, respectively, compared to metabolic syndrome rats which received HCHF and vehicle. ND: normal diet; HCHF: high-carbohydrate high-fat diet; VitaminC: vitamin C at a dose of 250 mg·kg^−1^ BW; Simvastatin: simvastatin at a dose of 1.3 mg·kg^−1^ BW; PMG50, PMG100, and PMG200: the phytosome containing the combined extracts of mulberry and ginger at a dose of 50, 100, and 200 mg·kg^−1^ BW, respectively.

Figure 5 shows the effect of PMG on the expression of PPAR-*γ* in adipose tissue. MetS rats induced by HCHF diet significantly decreased PPAR-*γ* expression in adipose tissue (*p* value < 0.001, compared to naïve control). However, all interventions used in this study including vitamin C, simvastatin, and PMG at all doses significantly increased PPAR-*γ* expression in adipose tissue (*p* value < 0.05, 0.01, 0.001, 0.001, and 0.001, respectively, compared to the HCHF diet-treated group).

### 3.7. Effect of PMG on Inflammatory Mediators

**Figure 6:**
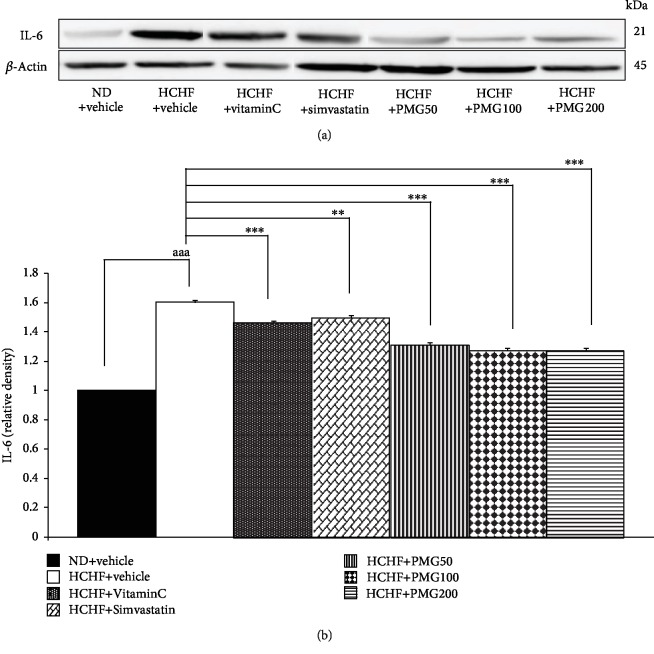
Effect of various doses of PMG on the expression of IL-6 in adipose tissue. (a) Representative western blot showing the levels of IL-6. (b) Relative density of IL-6. Data are presented as mean ± SEM (*n* = 6/group). ^aaa^*p* value < 0.001, compared to naïve control which received ND and vehicle and ^∗∗,∗∗∗^*p* value < 0.01 and 0.001, respectively, compared to metabolic syndrome rats which received HCHF and vehicle. ND: normal diet; HCHF: high-carbohydrate high-fat diet; VitaminC: vitamin C at a dose of 250 mg·kg^−1^ BW; Simvastatin: simvastatin at a dose of 1.3 mg·kg^−1^ BW; PMG50, PMG100, and PMG200: the phytosome containing the combined extracts of mulberry and ginger at a dose of 50, 100, and 200 mg·kg^−1^ BW, respectively.

**Figure 7:**
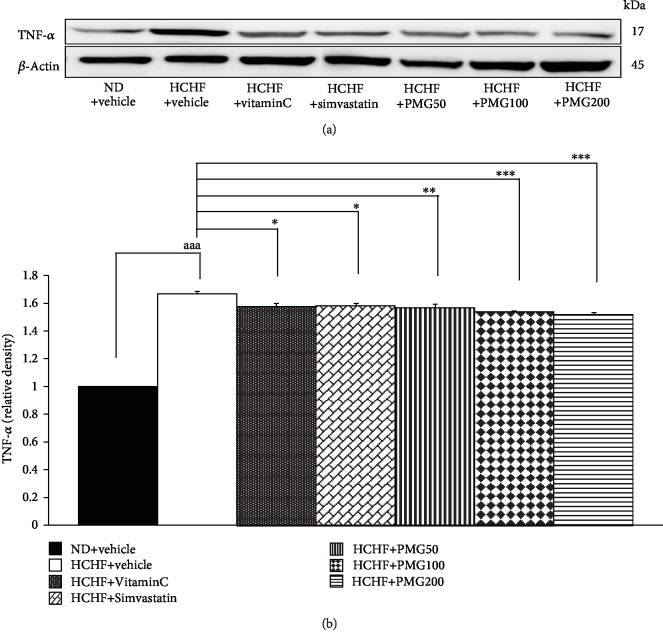
Effect of various doses of PMG on the expression of TNF-*α* in adipose tissue. (a) Representative western blot showing the levels of TNF-*α*. (b) Relative density of TNF-*α*. Data are presented as mean ± SEM (*n* = 6/group). ^aaa^*p* value < 0.001, compared to naïve control which received ND and vehicle and ^∗,∗∗,∗∗∗^*p* value < 0.05, 0.01, and 0.001, respectively, compared to metabolic syndrome rats which received HCHF and vehicle. ND: normal diet; HCHF: high-carbohydrate high-fat diet; VitaminC: vitamin C at a dose of 250 mg·kg^−1^ BW; Simvastatin: simvastatin at a dose of 1.3 mg·kg^−1^ BW; PMG50, PMG100, and PMG200: the phytosome containing the combined extracts of mulberry and ginger at a dose of 50, 100, and 200 mg·kg^−1^ BW, respectively.

Figures 6 and 7 show that HCHF diet increased IL-6 and TNF-*α* in adipose tissue (*p* value < 0.001 all, compared to naïve control). However, the changes of IL-6 and TNF-*α* were mitigated by vitamin C, simvastatin, and all doses of PMG treatments (*p* value < 0.001 and 0.05; *p* value < 0.01 and 0.05; *p* value < 0.001 and 0.01; *p* value < 0.001 all; *p* value < 0.001 all; compared to the HCHF diet-treated group).

## 4. Discussion

The current study has clearly revealed that PMG significantly improves most biological activities which related to MetS. The possible explanation may be associated with the reduction of active ingredients lost due to the encapsulation technique. PMG also improves numerous changes in MetS including the symptoms of MetS, AI index, HDAC3, PPAR-*γ*, adipose tissue, oxidative stress, and inflammation.

Since PPAR-*γ* serves as the major regulator of adipogenesis, the changes of both PPAR-*γ* and adipose tissue are also investigated. The current results show that MetS rats induced by HCHF diet significantly decrease PPAR-*γ* expression in adipose tissue and density of adipocyte but increase the size of adipocyte. The reduction of adipocyte density observed in this study may be associated with the reduction of PPAR-*γ* in adipose tissue. This finding are in agreement with the study of He and coworkers which demonstrates that targeted deletion of PPAR-*γ* in fat tissue induces the marked reduction in the number of adipocytes [35]. The possible explanation for the reduction of adipocyte density may occur partly via the adipogenesis reduction in preadipocyte [36] and apoptosis of adipocyte cell [37]. In addition to the reduction of adipocyte density, the adipocyte hypertrophy is also observed. It has been shown that targeted deletion of PPAR-*γ* in fat tissue induces the marked reduction in the number of adipocytes, alongside a compensatory hypertrophy of the remaining cells [35]. Therefore, the adipocyte hypertrophy observed in this study may occur partly via the compensatory hypertrophy of the remaining cells mentioned earlier. In addition, it may also occur as the result of the lipid sequestration of adipose tissue and the accumulation of triacylglyceride (TAG) in cytoplasmic lipid droplets (LDs) within adipocytes (fat cells) due to the caloric excess induced by MetS.

It has been revealed that adipocyte hypertrophy shows the positive correlation with insulin resistance (measured by HOMA-IR) and fasting plasma insulin in humans [38]. Therefore, insulin resistance and the elevation of plasma glucose in MetS rats may occur partly via adipocyte hypertrophy. However, PPAR-*γ* is also associated with gene regulating insulin sensitivity [39] and the regulation of glucose homeostasis via the decrease in gluconeogenesis and the increase in glycogen synthesis [40], so the reduction of PPAR-*γ* in adipose tissue may also play a role on both insulin resistance and the elevation of plasma glucose mentioned earlier. In addition, aforementioned effects, PPAR-*γ* also plays a role on lipid metabolism by decreasing triglyceride (TG) but increasing a high density of lipoprotein (HDL) [41]. Moreover, dyslipidemia observed in MetS also occurs as the results of adipocyte hypertrophy. Hypertrophic adipocyte induces the impairment of the process which incorporates FFAs into TGs leading to the decrease in FFA trapping and retention by adipose tissue which in turn gives rise to the elevation of FFA in plasma. This process that stimulates the triglyceride synthesis in the liver gives rise to the increase in VLDL and LDL but decreases HDL [42]. The dyslipidemia in MetS rats may also increase atherogenic index.

Under normal circumstance, PPAR-*γ* can also reverse macrophage infiltration in adipocyte and subsequently reduces inflammatory gene expression [43]. Therefore, the increase in the expressions of inflammatory cytokine such as IL-6 and TNF-*α* in adipose tissue in MetS rats may partly due to the decrease in PPAR-*γ*. PPAR-*γ* involves not only inflammation but also oxidative stress. PPARs also possesses anti-inflammatory and antioxidant properties by decreasing ROS production and upregulating the expression of antioxidant enzymes [44]. The current data also show the corresponding changes of oxidative stress markers. MetS rats showed the reduction of antioxidant enzymes such as SOD, CAT, and GSH-Px but increased the MDA level in adipose tissue from visceral and subcutaneous areas. The changes of oxidative stress markers just mentioned in serum also showed the same pattern.

MetS rats also demonstrate the high activity of angiotensin-converting enzyme (ACE) which indicates the stimulation of renin angiotensin system (RAS), a system playing an important role on the regulation of blood pressure [45]. The possible explanation for the stimulation of RAS resulting in the elevation of ACE may be associated with the secretion of aldosterone from adipocyte [46]. Therefore, the increased adipose tissue mass observed in MetS may possibly increase aldosterone which in turn stimulate the function of RAS resulting the increase ACE activity and finally increase angiotensin II resulting in hypertension.

Recent study has demonstrated that PPAR-*γ* function is regulated epigenetic modifications such as histone modification. The suppression of histone deacetylase 3 (HDAC3) can activate PPAR-*γ* expression [47]. Therefore, this information points out that the increase in HDAC3 in MetS rats may contribute the role on the reduction in PPAR-*γ* expression in adipose tissue especially at a high dose of the phytosome containing the combined extract of mulberry and ginger. This change in turn induces the increases in oxidative stress status, inflammation, dyslipidemia, insulin resistance, and hyperglycemia. In addition, it is also responsible for the increase adiposity, hypertrophic adipocyte. However, the current results show that HDAC3 failed to show the closed relationship with PPAR-*γ*. Therefore, other factors such as other modification processes of histone, DNA, and other factors influencing the transcription and translation processes may also play the role on the regulation of PPAR-*γ*.

However, the aforementioned changes can be improved by vitamin C, simvastatin, and all doses of phytosome containing the combined extract of mulberry and ginger. The possible underlying mechanism may occur partly via the increase in PPAR-*γ* expression in adipose tissue which in turn improves oxidative stress status, inflammation, dyslipidemia, insulin resistance, and hyperglycemia together with the reduction of adipose tissue in MetS rats. Vitamin C also exerts the positive modulation partly via the increase in PPAR-*γ* expression in adipose tissue. However, other mechanisms such as the alterations in adipokines may also contribute the role and required further investigation. Based on the activation effect of anthocyanins on PPAR-*γ* [48, 49], we suggest that the positive modulation effect of phytosome containing the combined extract of mulberry and ginger may be associated with anthocyanins [8] and gingerol [50].

## 5. Conclusion

**Figure 8:**
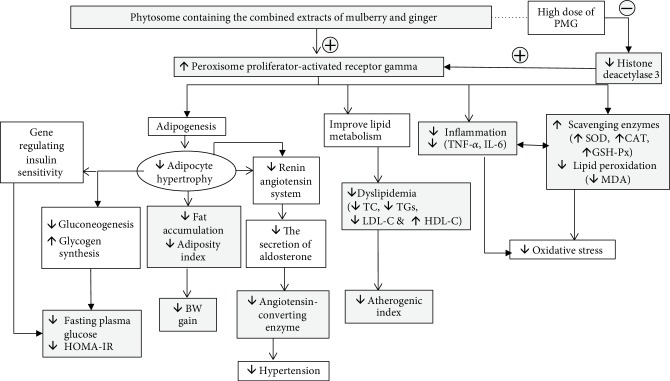
The schematic diagram demonstrated the positive modulation effect of PMG in the animal model of metabolic syndrome. HOMA-IR: homeostasis model assessment of insulin resistance; TC: total cholesterol; TGs: triglyceride; LDL-C: low-density lipoprotein cholesterol; HDL-C: high-density lipoprotein cholesterol; BW: body weight; TNF-*α*: tumor necrosis factor-*α*; IL-6: interleukin-6; SOD: superoxide dismutase; CAT: catalase; GSH-Px: glutathione peroxidase; MDA: malondialdehyde.

Our study has clearly demonstrated that phytosome containing the combined extract of mulberry and ginger can improve MetS. The possible underlying mechanism occurs via the multipathway including the increase in PPAR-*γ* which in turn decreases adipose tissue, dyslipidemia, insulin resistance, inflammation, and oxidative stress. The epigenetic modification also plays the role especially at a high dose of the phytosome containing the combined extract of mulberry and ginger as shown in Figure 8. Therefore, this product can be served as the potential supplement to manage MetS. However, a clinical trial study is essential to confirm this health benefit.

## Figures and Tables

**Table 1:** Characterization of PMG including phenolic compositions and biological activities.

Parameters	Units	EMG	PMG	Standard reference
Total phenolic	mg GAE/mg extract	243.00 ± 30.55	273.00 ± 5.77	—
Total flavonoids	*μ*g quercetin/mg extract	90.33 ± 15.40	137.00 ± 3.85^∗^	—
Gingerol	*μ*g gingerol/50 mg extract	9.45 ± 0.03	19.23±0.03^∗∗∗^	—
Cyanidin-3-O-glucoside	*μ*g Cyn-3-glu/50 mg extract	11.63 ± 0.06	16.01±0.06^∗∗∗^	—
Quercetin-3-rutinoside	*μ*g Rutin/50 mg extract	25.93 ± 0.17	39.82±0.40^∗∗^	—
Ferulic acid	*μ*g ferulic acid/50 mg extract	12.89 ± 0.42	20.00 ± 0.18^∗^	—
Gallic acid	*μ*g GAE/50 mg extract	11.26 ± 0.01	47.89±0.16^∗∗∗^	—
Antioxidant activities				
DPPH	EC 50 (mg/ml)	39.97 ± 1.68	39.67 ± 0.93	0.03 ± 0.01, ascorbic acid
FRAP	EC 50 (mg/ml)	97.15 ± 3.17	41.28±0.37^∗∗∗^	122.19 ± 12.82, ascorbic acid
ABTS	EC 50 (mg/ml)	58.46 ± 0.66	47.28 ± 1.89^∗^	0.20 ± 0.002, trolox
Obesity marker				
Pancreatic lipase	EC 50 (mg/ml)	181.56 ± 17.87	138.15 ± 0.92^∗^	0.002 ± 0.001, orlistat
Inflammatory marker				
COX-II	EC 50 (mg/ml)	81.25 ± 2.97	80.42 ± 1.85	0.02 ± 0.001, indomethacin

Data are presented as mean ± SEM. ^∗,∗∗,∗∗∗^*p* value < 0.05, 0.01, and 0.001, respectively, compared between EMG and PMG. EMG: the combined extracts of mulberry and ginger, PMG: phytosome containing the combined extracts of mulberry and ginger.

**Table 2:** The effect of various doses of PMG on metabolic parameters.

Parameters	ND+vehicle	HCHF+vehicle	HCHF+VitaminC	HCHF+Simvastatin	HCHF+PMG50	HCHF+PMG100	HCHF+PMG200
Body weight gain (%)	3.94 ± 0.44	6.71 ± 0.03^a^	3.49 ± 1.01	2.33±0.75^∗∗^	−5.47±0.55^∗∗∗^	−7.89±0.45^∗∗∗^	−6.42±1.18^∗∗∗^
Cholesterol (mg/dl)	68.20 ± 3.32	100.20 ± 2.80^aa^	85.20 ± 6.40	74.60 ± 4.95^∗^	73.60±1.57^∗∗^	69.20±2.03^∗∗^	69.00±3.48^∗∗^
Triglyceride (mg/dl)	72.80 ± 3.25	112.00 ± 1.41^aaa^	101.00 ± 5.43	75.00±2.35^∗∗∗^	75.80±3.80^∗∗∗^	75.80±4.31^∗∗∗^	73.60±2.54^∗∗∗^
LDL-C (mg/dl)	28.20 ± 1.16	65.00 ± 3.08^aaa^	58.80 ± 1.69	31.00±3.08^∗∗∗^	33.00±1.58^∗∗∗^	33.00±2.00^∗∗∗^	32.40±1.63^∗∗∗^
HDL-C (mg/dl)	59.40 ± 1.89	38.80 ± 1.53^aa^	44.60 ± 1.72	54.60 ± 2.42^∗^	53.80 ± 3.29^∗^	54.60 ± 2.48^∗^	54.80 ± 4.91^∗^
Atherogenic index	1.16 ± 0.08	2.61 ± 0.16^aaa^	1.94 ± 0.23	1.51±0.04^∗∗^	1.05±0.06^∗∗∗^	1.00±0.12^∗∗∗^	1.04±0.08^∗∗∗^
Plasma glucose AUC (mg h/dl)	107.17 ± 3.88	207.33 ± 25.98^aa^	184.50 ± 5.72	201.17 ± 16.83	144.67 ± 5.20	137.17 ± 7.13	127.50 ± 5.59^∗^
HOMA-IR	2.13 ± 0.09	3.36 ± 0.18^aaa^	3.23 ± 0.19	3.01 ± 0.08	2.57 ± 0.08^∗^	2.47±0.09^∗∗^	2.57 ± 0.08^∗^
ACE (units/mg protein)	0.03 ± 0.00	0.14 ± 0.00^aaa^	0.10 ± 0.00	0.08 ± 0.00^aa,^^∗∗^	0.05±0.00^∗∗∗^	0.05±0.01^∗∗∗^	0.05±0.00^∗∗∗^

Data are presented as mean ± SEM (*n* = 6/group). ^a, aa, aaa^*p* value < 0.05, 0.01, and 0.001, respectively, compared to naïve control which received ND and vehicle and ^∗,∗∗,∗∗∗^*p* value < 0.05, 0.01, and 0.001, respectively, compared to metabolic syndrome rats which received HCHF and vehicle. ND: normal diet; HCHF: high-carbohydrate high-fat diet; VitaminC: vitamin C at a dose of 250 mg·kg^−1^ BW; Simvastatin: simvastatin at a dose of 1.3 mg·kg^−1^ BW; PMG50, PMG100, and PMG200: the phytosome containing the combined extracts of mulberry and ginger at a dose of 50, 100, and 200 mg·kg^−1^ BW, respectively.

**Table 3:** The effect of various doses of PMG on adipose tissue alterations.

Parameters		ND+vehicle	HCHF+vehicle	HCHF+VitaminC	HCHF+Simvastatin	HCHF+PMG50	HCHF+PMG100	HCHF+PMG200
Size of adipocyte (*μ*m)	Visceral area	46.10 ± 1.00	84.79 ± 1.31^aaa^	81.46 ± 5.25	78.82 ± 1.56^∗^	64.68±0.99^∗∗∗^	63.30±1.53^∗∗∗^	53.04±1.38^∗∗∗^
	Subcutaneous area	33.37 ± 0.97	55.25 ± 1.50^aaa^	51.29 ± 0.87	51.00 ± 1.24	48.50±1.43^∗∗^	48.38±2.12^∗∗^	43.20±1.90^∗∗∗^

Density of adipocyte (adipocytes/225 *μ*m^2^)	Visceral area	62.33 ± 0.99	25.50 ± 0.43^aaa^	26.33 ± 0.76	30.33±0.99^∗∗^	37.67±0.42^∗∗∗^	38.50±0.56^∗∗∗^	47.32±0.61^∗∗∗^
	Subcutaneous area	56.50 ± 1.45	30.33 ± 2.07^aaa^	33.50 ± 1.80	32.33 ± 2.16	36.33 ± 1.74^∗^	40.33±0.99^∗∗∗^	43.67±4.72^∗∗∗^

Adiposity index (AI) (%)	Visceral area	3.35 ± 0.21	5.85 ± 0.18^aaa^	5.60 ± 0.18	5.06 ± 0.25	5.92 ± 0.09	3.49±0.43^∗∗∗^	3.14±0.08^∗∗∗^
	Subcutaneous area	3.42 ± 0.18	3.74 ± 0.15	3.54 ± 0.11	3.68 ± 0.09	3.60 ± 0.15	3.38 ± 0.12	3.16 ± 0.13

Weights of white adipose tissues (g/kg BW)	Visceral area	14.59 ± 0.84	32.29 ± 0.84^aaa^	30.86 ± 1.09	30.87 ± 0.83	30.09 ± 0.70	16.92±0.86^∗∗∗^	15.52±0.33^∗∗∗^
	Subcutaneous area	14.91 ± 0.86	19.22 ± 1.00^aa^	19.51 ± 0.56	18.79 ± 0.93	18.26 ± 0.56	16.36 ± 0.52^∗^	15.62±0.50^∗∗^

Data are presented as mean ± SEM (*n* = 6/group). ^aa, aaa^*p* value < 0.01 and 0.001, respectively, compared to naïve control which received ND and vehicle and ^∗,∗∗,∗∗∗^*p* value < 0.05, 0.01 and 0.001, respectively, compared to metabolic syndrome rats which received HCHF and vehicle. ND; normal diet; HCHF: high-carbohydrate high-fat diet; VitaminC: vitamin C at a dose of 250 mg·kg^−1^ BW; Simvastatin: simvastatin at a dose of 1.3 mg·kg^−1^ BW; PMG50, PMG100, and PMG200: the phytosome containing the combined extracts of mulberry and ginger at a dose of 50, 100, and 200 mg·kg^−1^ BW, respectively.

**Table 4:** The effect of various doses of PMG on oxidative stress markers in the visceral area.

Treatment group	MDA (ng/mg protein)	SOD (units/mg protein)	CAT (units/mg protein)	GSH-Px (units/mg protein)
ND+vehicle	0.54 ± 0.05	16.52 ± 0.81	69.77 ± 3.98	17.67 ± 0.67
HCHF+vehicle	2.97 ± 0.15^aaa^	4.11 ± 0.24^aaa^	26.13 ± 1.66^aaa^	4.37 ± 0.36^aaa^
HCHF+VitaminC	1.14±0.14^∗∗∗^	9.74±0.79^∗∗∗^	48.52±4.37^∗∗^	10.87±0.83^∗∗∗^
HCHF+Simvastatin	2.46 ± 0.07^∗^	5.93±0.49^∗∗∗^	36.53 ± 2.45	5.13 ± 0.38
HCHF+PMG50	0.64±0.06^∗∗∗^	9.94±0.49^∗∗∗^	41.92 ± 2.34	10.57±0.83^∗∗∗^
HCHF+PMG100	0.69±0.08^∗∗∗^	9.21±0.57^∗∗∗^	45.34 ± 2.50^∗^	10.65±0.64^∗∗∗^
HCHF+PMG200	0.51±0.03^∗∗∗^	8.01±0.84^∗∗^	45.40 ± 3.22^∗^	10.77±1.27^∗∗∗^

Data are presented as mean ± SEM (*n* = 6/group). ^aaa^*p* value < 0.001, compared to naïve control which received ND and vehicle and ^∗,∗∗,∗∗∗^*p* value < 0.05, 0.01, and 0.001, respectively, compared to metabolic syndrome rats which received HCHF and vehicle. ND: normal diet; HCHF: high-carbohydrate high-fat diet; VitaminC: vitamin C at a dose of 250 mg·kg^−1^ BW; Simvastatin: simvastatin at a dose of 1.3 mg·kg^−1^ BW; PMG50, PMG100, and PMG200: the phytosome containing the combined extracts of mulberry and ginger at a dose of 50, 100, and 200 mg·kg^−1^ BW, respectively.

**Table 5:** The effect of various doses of PMG on oxidative stress markers in the subcutaneous area.

Treatment group	MDA (ng/mg protein)	SOD (units/mg protein)	CAT (units/mg protein)	GSH-Px (units/mg protein)
ND+vehicle	0.34 ± 0.05	7.14 ± 0.23	77.91 ± 5.09	11.38 ± 0.37
HCHF+vehicle	1.70 ± 0.26^aaa^	3.04 ± 0.22^aaa^	40.11 ± 1.45^aaa^	1.64 ± 0.19^aaa^
HCHF+VitaminC	0.60±0.04^∗∗∗^	5.94 ± 0.54^∗^	63.59±3.51^∗∗^	7.53±0.55^∗∗∗^
HCHF+Simvastatin	1.31 ± 0.13	3.66 ± 0.43	46.07 ± 4.13	4.26 ± 0.34
HCHF+PMG50	0.40±0.02^∗∗∗^	6.25±0.24^∗∗∗^	62.35±5.41^∗∗^	6.87±0.75^∗∗∗^
HCHF+PMG100	0.36±0.04^∗∗∗^	6.65±0.74^∗∗∗^	64.95±3.72^∗∗^	7.18±0.54^∗∗∗^
HCHF+PMG200	0.39±0.02^∗∗∗^	6.43±0.56^∗∗∗^	63.38±4.54^∗∗^	7.52±0.66^∗∗∗^

Data are presented as mean ± SEM (*n* = 6/group). ^aaa^*p* value < 0.001, compared to naïve control which received ND and vehicle and ^∗,∗∗,∗∗∗^*p* value < 0.05, 0.01, and 0.001, respectively, compared to metabolic syndrome rats which received HCHF and vehicle. ND: normal diet; HCHF: high-carbohydrate high-fat diet; VitaminC: vitamin C at a dose of 250 mg·kg^−1^ BW; Simvastatin: simvastatin at a dose of 1.3 mg·kg^−1^ BW; PMG50, PMG100, and PMG200: the phytosome containing the extracts of mulberry and ginger at a dose of 50, 100, and 200 mg·kg^−1^ BW, respectively.

**Table 6:** The effect of various doses of PMG on oxidative stress markers in serum.

Treatment group	MDA (ng/mg protein)	SOD (units/mg protein)	CAT (units/mg protein)	GSH-Px (units/mg protein)
ND+vehicle	0.28 ± 0.04	7.14 ± 0.23	75.34 ± 2.51	10.14 ± 0.54
HCHF+vehicle	1.58 ± 0.23^aaa^	1.25 ± 0.43^aaa^	23.50 ± 6.85^aaa^	1.48 ± 0.53^aaa^
HCHF+VitaminC	0.49±0.02^∗∗∗^	4.58±0.30^∗∗∗^	55.72 ± 6.74	5.03 ± 0.42^∗^
HCHF+Simvastatin	1.16 ± 0.12^∗^	3.66 ± 0.43^∗^	39.97 ± 4.30	3.79 ± 0.30
HCHF+PMG50	0.35±0.02^∗∗∗^	6.26±0.24^∗∗∗^	62.85±7.71^∗∗∗^	7.79±0.46^∗∗∗^
HCHF+PMG100	0.31±0.04^∗∗∗^	6.66±0.74^∗∗∗^	71.57±8.25^∗∗∗^	7.93±1.14^∗∗∗^
HCHF+PMG200	0.31±0.02^∗∗∗^	6.43±0.56^∗∗∗^	72.73±5.12^∗∗∗^	7.59±0.31^∗∗∗^

Data are presented as mean ± SEM (*n* = 6/group). ^aaa^*p* value < 0.001, compared to naïve control which received ND and vehicle and ^∗,∗∗∗^*p* value < 0.05 and 0.001, respectively, compared to metabolic syndrome rats which received HCHF and vehicle. ND: normal diet; HCHF: high-carbohydrate high-fat diet; VitaminC: vitamin C at a dose of 250 mg·kg^−1^ BW; Simvastatin: simvastatin at a dose of 1.3 mg·kg^−1^ BW; PMG50, PMG100, and PMG200: the phytosome containing the combined extracts of mulberry and ginger at a dose of 50, 100, and 200 mg·kg^−1^ BW, respectively.

## Data Availability

I confirm that data are available and will be provided on request because during this period, all data are in the process of petty patent registration.
