# Correction to “Antimetabolic Syndrome Effect of Phytosome Containing the Combined Extracts of Mulberry and Ginger in an Animal Model of Metabolic Syndrome”

**DOI:** 10.1155/omcl/9871678

**Published:** 2025-11-21

N. Palachai, J. Wattanathorn, S. Muchimapura, W. Thukham-mee, “Antimetabolic Syndrome Effect of Phytosome Containing the Combined Extracts of Mulberry and Ginger in an Animal Model of Metabolic Syndrome,” *Oxidative Medicine and Cellular Longevity*, 2019, https://doi.org/10.1155/2019/5972575.

In the article, the units used to describe the total phenolic values are incorrect, where “mg GAE/mg extract” should read “µg GAE/mg extract.”

This error exists in the first row, second column of Table 1, and in Sections 2.3 and 3.1. Specifically:

1. “Result was expressed mg gallic acid equivalent (GAE)/mg extract.”
  should read:
  “Result was expressed μg gallic acid equivalent (GAE)/mg extract.”
2. “It was found that EMG contained total phenolic and flavonoid contents at the concentrations of 243.00 ± 30.55 mg GAE/mg extract and 90.33 ± 15.40 μg quercetin/mg extract, respectively.”
  should read:
  “It was found that EMG contained total phenolic and flavonoid contents at the concentrations of 243.00 ± 30.55 µg GAE/mg extract and 90.33 ± 15.40 μg quercetin/mg extract, respectively.”
3. “However, the contents of total phenolic, flavonoids, gingerol, cyanidin-3-O-glucoside, quercetin-3-rutinoside, ferulic acid, and gallic acid in PMG were 273.00 ± 5.77 mg GAE/mg extract, 137.00 ± 3.85 µg quercetin/mg extract, 19.23 ± 0.03 µg gingerol/50 mg extract, 16.01 ± 0.06 µg Cyn-3-glu/50 mg extract, 39.82 ± 0.40 µg Rutin/50 mg extract, 20.00 ± 0.18 µg ferulic acid/50 mg extract, and 47.89 ± 0.16 µg GAE/50 mg extract, respectively.”
  should read:
4. “However, the contents of total phenolic, flavonoids, gingerol, cyanidin-3-O-glucoside, quercetin-3-rutinoside, ferulic acid, and gallic acid in PMG were 273.00 ± 5.77 µg GAE/mg extract, 137.00 ± 3.85 µg quercetin/mg extract, 19.23 ± 0.03 µg gingerol/50 mg extract, 16.01 ± 0.06 µg Cyn-3-glu/50 mg extract, 39.82 ± 0.40 µg Rutin/50 mg extract, 20.00 ± 0.18 µg ferulic acid/50 mg extract, and 47.89 ± 0.16 µg GAE/50 mg extract, respectively.”

The authors apologize for these errors.

